# Case report: a case report and literature analysis on intestinal tuberculosis intestinal perforation complicated by umbilical intestinal fistula and bladder ileal fistula

**DOI:** 10.1186/s12879-023-08550-z

**Published:** 2023-08-28

**Authors:** Guobin Liu, Tianyan Chen, Xiaofeng Song, Bolin Chen, Quan Kang

**Affiliations:** Department of General Surgery and Trauma Surgery, Ministry of Education Key Laboratory of Child Development and Disorders, National Clinical Research Center for Child Health and Disorders, International Science and Technology Cooperation Base of Child Development and Critical Disorders, Chongqing Key Laboratory of Pediatrics, Children’s Hospital of Chongqing Medical University, 400014 Chongqing, China

**Keywords:** Intestinal tuberculosis, Intestinal perforation, Umbilical intestinal fistula, Bladder ileal fistula

## Abstract

**Background:**

Intestinal tuberculosis is a chronic and specific infection caused by Mycobacterium tuberculosis invading the intestine. Due to the nonspecific clinical presentation, it is stressed that intestinal perforation complicates umbilical intestinal fistula and bladder ileal fistula is very rare and extremely difficult to be diagnosed. It is significant to identify the disease and take urgent intervene in the early stage.

**Case presentation:**

An 18-month-old boy patient presented with abdominal pain. Abdominal CT suggested abscess formation in the right lower abdomen and pelvis. The patient underwent resection of necrotic and stenotic intestinal segments with the creation of an ileostomy, cystostomy and vesicoureteral fistula repair for the presence of intestinal perforation complicated by vesicoureteral fistula and umbilical enterocutaneous fistula. Histopathology confirmed the intestinal tuberculosis. The patient was discharged successfully after 11 days post anti-tuberculosis treatment.

**Conclusion:**

Our case report here is a rare case of umbilical intestinal fistula with bladder ileal fistula secondary to intestinal perforation from intestinal tuberculosis. The purpose of this report is to make the surgical community aware of atypical presentations of intestinal tuberculosis. If our peers encounter the similar situation, they can be prepared for corresponding diagnosis and treatment.

## Introduction

Tuberculosis (TB) remains a significant global problem, with an estimated 10.6 million people infected and 1.6 million deaths worldwide in 2021 [1]. Of these, Children account for 11% of all tuberculosis cases and 14% of all tuberculosis -related deaths [2].

Intestinal tuberculosis is a chronic specific infection caused by the invasion of Mycobacterium tuberculosis into the intestine, accounting for 1–3% of all TB cases [3]. Intestinal tuberculosis can lead to serious complications such as intestinal obstruction, perforation, intestinal fistula, intra-abdominal effusion, and gastrointestinal bleeding. The clinical manifestations of intestinal tuberculosis are not obvious, making it difficult to be diagnosed and differentiated from other diseases as well as prone to misdiagnosis.

Here we report a rare case of umbilical enteric fistula and bladder ileal fistula caused by intestinal perforation secondary to intestinal tuberculosis, which was a diagnostic challenge and can only be diagnosed after surgery.

## Case report

**Fig. 1:**
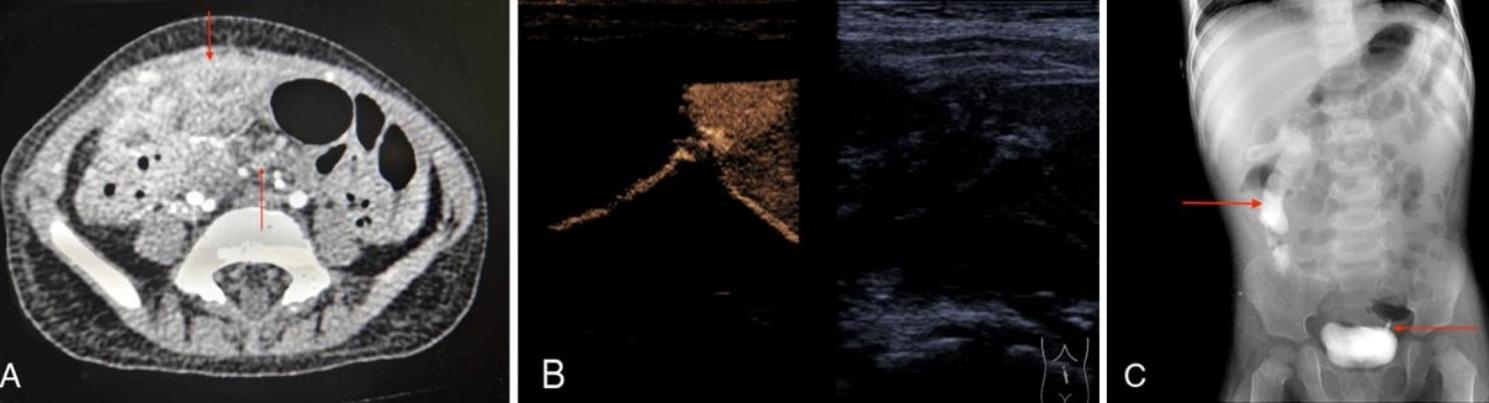
Preoperative image examinations

The patient was an 18-month-old boy, 8.4 kg (2.4 SD lower than peers), who presented with paroxysmal abdominal pain with low-grade fever and diarrhea for 9 days. Physical examination revealed that the patient had a slightly distended abdomen with scattered pressure pain throughout the abdomen, especially in the right lower abdomen. It has a mass with a diameter of 6 cm located in his lower right abdomen. By inquiring about the family medical history, it was known that the grandfather of the patient died due to TB one year ago, and the patient lived with his grandfather. Following, comprehensive laboratory tests had been conducted which included the following index: white blood cell count 18.15 (×10^9/L) ↑, hemoglobin 76 (g/L) ↓, lymphocyte percentage 30.1 (%) ↓, C-reactive protein 30.89 (mg/L) ↑; IL-8 55.84 (pg/mL) ↑; HIV negative; urine routine: red blood cells 48 (pcs/µL) ↑, white blood cells 724 (pcs/µL) ↑, Pus cell mass 39(pcs/µL) ↑; stool routine: leukocytes 3–6(pcs/HP) ↑, positive occult blood test. Abdominal ultrasound suggested a fluid-containing lesion in the right lower abdomen with a viscous composition. Chest X-ray: No obvious abnormality; abdominal X-ray suggested scattered inflation of the small intestine, mainly in the left abdomen. Abdominal CT (Fig. 1A) indicated abnormal images of the right lower abdomen and pelvis; the intestinal wall was observed more thickened with enhancement. There was the possibility of infectious lesions and abscess formation. The admission diagnosis was considered a peri appendiceal abscess. After admission, the patient was given suspended erythrocytes 0.25U to correct anemia. Symptomatic treatment had been done by employing ceftizoxime and metronidazole for anti-infection and correction of water-electrolyte disturbance, respectively. On day 7 after admission, the patient showed a “fecal-like substance” in his urine. Laboratory tests included: urine routine: bilirubin 17(µmol/L) ↑, protein 0.15(g/L) ↑, erythrocytes 241(g/µL) ↑, leukocytes 709(g/µL) ↑. The urine culture was negative; Gram-positive cocci were found on the urine smear; the urine smear did not show acid-fast bacilli. On day 10, the patient had redness and swelling in the umbilicus with visible secretions. Ultrasound images of the abdomen and umbilicus (Fig. 1B) showed that mucus composition was observed in the right abdomen, with slow intestinal peristalsis. The partial intestinal dilatation was still visible on the right side of the abdomen; a fluid-containing inflammatory lesion with mucous composition can be seen in the umbilicus, which extended deeper and was seen to be connected to an abscess in the abdominal cavity. On day 14, the patient’s umbilicus was ruptured with visible purulent secretions, and there were still many fecal-like substances in the urine. Retrograde urographic findings (Fig. 1C) showed that the contrast agent entered the intestinal cavity from the bladder, suggesting the bladder was connected to the intestine, furthermore, ureteral reflux was seen on the left side of the bladder.

A) Abdominal CECT: The upper arrow indicates suspicious lesions of the bowel and the lower arrow indicates small mesangial nodules.
B) Umbilical ultrasound: a liquid inflammatory lesion in the umbilical cord, with a thick component, the lesion extends deeply, and it can be seen to communicate with the intra-abdominal abscess.
C) Urography: Bladder filling and round-like translucent shadows were observed; The upper arrow indicates contrast is seen in the right abdominal part of the bowel tube, indicating that the bladder may communicate with the intestine; The lower arrow indicates signs of bladder-left ureteral reflux.

**Fig. 2:**
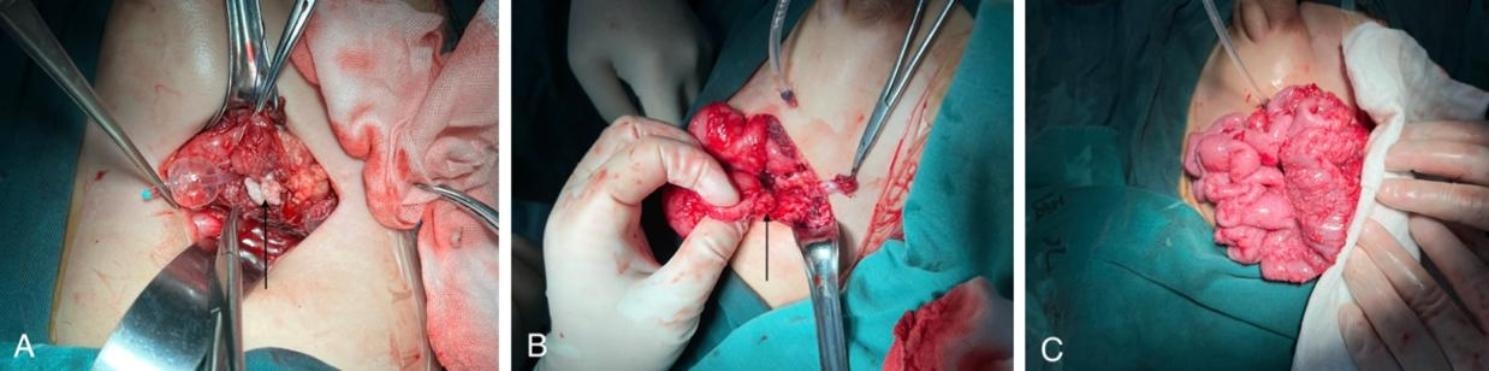
Intraoperative images

On the 15th day of hospitalization, the patient underwent surgical intervention (Fig. 2). Under anesthesia, cystoscopy revealed the presence of a fistula at the base of the bladder, prompting further exploration using laparoscopy. Extensive intra-abdominal adhesions were encountered, posing challenges to the surgical dissection, necessitating conversion to open surgery. In the lower right abdomen, a pelvic abscess and miliary granulations were observed. The abscess cavity was meticulously debrided, uncovering an ileal perforation with an associated bladder fistula. To establish bladder diversion, an inflatable catheter was placed in the anterior bladder wall, followed by repair of the bladder defect. Following adhesiolysis, necrotic and partially stenotic segments of the distal ileum were identified, warranting the performance of an ileostomy. Resection of the non-viable bowel segments was performed, and the distal ileum was securely anastomosed. Additionally, an appendectomy was carried out. Thorough irrigation of the abdominal cavity was performed, and a drainage tube was placed for effective drainage. Subsequently, the patient was transferred to the intensive care unit for postoperative management.

A) Bladder wall necrosis is seen, and arrows indicate caseous tissue in the bladder.
B) The arrow indicates the perforation of the ileum.
C) Diffuse distribution of white miliary-like particles on the serous membrane is visible.

**Fig. 3:**
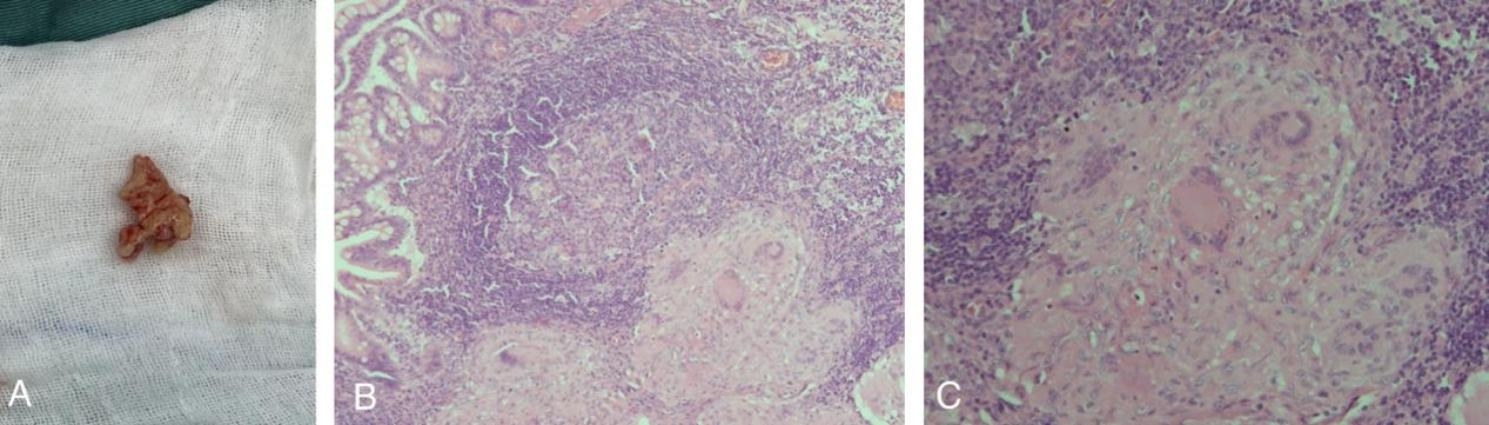
Pathological analysis of ileal mucosal nodules by H&E stain

Based on the above findings, it was suggested that the possibility of abdominal tuberculosis in this patient. Thus, further tests were performed including tuberculosis interferon test, gastric fluid and sputum antacid bacillus smear, gastric fluid and sputum tuberculosis culture, and sputum x-pert all with positive results. Subsequently, pathological findings confirmed abdominal tuberculosis infection by H&E stain (Fig. 3). The patient was then transferred to the infection unit to receive continuous anti-tuberculosis (isoniazid 100 mg qd, rifampicin 125 mg qd, linezolid 85 mg tid), intravenous nutrition, and other symptomatic treatments, the patient’s condition was stabilized, and he was successfully discharged from the hospital on the 11th-day post-anti-tuberculosis treatment.

A) Image of small nodules of the ileal mucosa.
B) Image of a granuloma of the mucosa (100-fold).
C) Image of a granuloma of the mucosa (200-fold).

We explained the very rare pathophysiology of intestinal tuberculosis with intestinal perforation complicated by umbilical intestinal fistula and bladder ileal fistula to the patient’s guardian and obtained informed consent for the case to be published in a medical journal.

## Discussion

Intestinal tuberculosis is a chronic and specific infection caused by Mycobacterium tuberculosis invading the intestine, with abdominal pain, bloating, and weight loss as the primary clinical manifestations [4]. In infants, this usually occurs through inhalation or ingestion of the mother’s respiratory droplets or through contact with infected breast tissue [5].

Primary abdominal tuberculosis refers to the direct invasion of abdominal organs and tissues by Mycobacterium tuberculosis during the initial infection, commonly seen in children and immunocompromised individuals. Secondary abdominal tuberculosis, on the other hand, refers to the spread of tuberculous lesions to the abdomen from other sites through the bloodstream or lymphatic system, typically observed in adults and individuals with normal immune function [6]. The patient, 18 months old, had a family history of tuberculosis. Postoperative pathology confirmed abdominal tuberculosis infection. Chest X-ray did not show any abnormalities. Acid-fast bacilli smear, sputum culture for tuberculosis, and X-pert MTB/RIF assay of the sputum all yielded negative results, suggesting primary abdominal tuberculosis.

Intestinal tuberculosis can lead to severe complications, and a study on intestinal tuberculosis showed a complication rate of 44% (27/61) in 2017, including the formation of abscesses, fistulas, strictures, perforations, and obstructions [7]. Abdominal symptoms, as the first symptom, is a risk factor for intestinal perforation complicated by abdominal tuberculosis [8]. The ileocecal region, due to its unique anatomical structure and high absorption rate, is a common site for intestinal perforation in cases of intestinal tuberculosis [9]. The incidence of intestinal tuberculosis in the ileocecal region is approximately 17–42% [10, 11]. In this region, caseous necrosis and abscess formation are commonly observed. The rupture of these abscesses can lead to the dissemination of tuberculosis bacilli to surrounding tissues and organs. One possible route of dissemination is the entry of tuberculosis bacilli into the bladder, resulting in the development of secondary bladder tuberculosis. Bladder tuberculosis typically occurs because of renal tuberculosis, and it is less commonly associated with intestinal tuberculosis. In this scenario, bladder tuberculosis can potentially lead to complications such as enterovesical fistula and bladder perforation [12].

Diagnosing intestinal tuberculosis is currently a challenge. The insidious onset and nonspecific clinical presentation of intestinal tuberculosis and the lack of expressive skills in children lead to frequent underdiagnosis and misdiagnosis. Therefore, we must combine clinical manifestations, laboratory tests, pathogenic tests, and imaging tests before making a diagnosis.

The clinical symptoms of intestinal tuberculosis are nonspecific, mostly presenting as chronic abdominal pain, and acute abdominal pain is mainly associated with complications [13]. W. Cheng et al. conducted a retrospective study of 85 patients with intestinal tuberculosis. They found abdominal pain in 75 cases (88.2%), of which 21 cases were acute abdominal pain due to intestinal perforation. The remaining 54 cases presented with Chronic pain around the umbilicus and right lower abdomen [14]. Patients with intestinal tuberculosis may present with weight loss accompanied by mild to moderate anemia due to various reasons such as chronic inflammatory abscesses, reduced intake, and impaired absorption. Other frequent gastrointestinal symptoms include chronic diarrhea, constipation, and decreased appetite. On physical examination, ascites and palpable abdominal masses are often found, especially in the right lower abdominal region (19.3%) and splenomegaly (14.2%) [13].

patients with intestinal tuberculosis usually present with elevated ESR, mild to moderate anemia, hypoalbuminemia, and leukocytosis [15]. W. Cheng et al. concluded that PPD has a high diagnostic value in patients not vaccinated against TB. At the same time, ESR is useful in assessing efficacy, and T-SPOT has a specificity of 92% for TB [14]. IGRA is highly specific for diagnosing latent tuberculosis infection (LTBI), especially in those who received BCG vaccination [16]. Continuous CRP values are useful in assessing the response of abdominal tuberculosis to anti-tuberculosis therapy. The absence of a decrease in CRP levels may suggest other diagnoses or drug-resistant TB [17].

Antacid bacillus staining and Mycobacterium tuberculosis culture are widely used in the diagnosis of intestinal tuberculosis, and it is recommended that this test be routinely performed in patients with intestinal tuberculosis as an indicator to assess response to treatment. Mycobacterium tuberculosis culture is the gold standard for diagnosing intestinal TB, especially for patients who will undergo a colonoscopy and have collected tissue specimens [13].

Using ultrasound as a diagnostic tool to look for specific features of abdominal tuberculosis is less reliable because of its high false-negative rate and the subjective nature of the manipulation and interpretation of the results, which tend to miss subtle signs [18]. CT can detect changes within the intestinal wall and complications such as obstruction and perforation, making it an excellent diagnostic tool for intestinal tuberculosis. Intestinal tuberculosis in CECT may present as circumferential wall thickening and increased enhancement of terminal ileum, asymmetric thickening of the ileocecal valve, strictures in distal ileum with upstream bowel loop dilation, and necrotic enlarged lymph nodes in the draining area [19].

Endoscopy plays an important role in diagnosis by complementing other modalities. it may be the initial tool for diagnosing different symptoms and presentations. An additional benefit of endoscopy is obtaining specimens for histopathologic and microbiologic analysis [15].

In cases of diagnostic uncertainty, a surgical approach through laparoscopy or dissection may increase the chances of early diagnosis [4]. Diagnostic laparoscopic exploration combined with tissue biopsy is the gold standard for diagnosing peritoneal tuberculosis, and the typical laparoscopic presentation of peritoneal tuberculosis is: (1) Multiple yellow-white tubercles scattered all over the visceral and parietal peritoneum; (2) Omental thickening with ascites; (3) Fibrous bands extending from parietal peritoneum to visceral peritoneum; (4) Abdominal cocoon with matted small bowel [20].

Intestinal tuberculosis is characterized by chronic granulomatous inflammation in the gastrointestinal tract, a collection of vaguely contoured epithelioid histiocytes (macrophages), usually large (> 200 μm), confluent, dense (> 5–10/hpf), submucosa, characterized by central caseous changes, which is diagnostic for ITB. Other features commonly seen in ITB include submucosal granulomas, ulcers lined with epithelioid histiocytes, and disproportionate submucosal inflammation [21]. In our case, the child presented with non-specific abdominal pain symptoms. The diagnosis of a peri appendiceal abscess was considered on admission according to the ultrasound image. He was easily misdiagnosed with peri appendiceal abscess because of the high clinical similarity between the child’s presentation and peri appendiceal abscess.

The treatment approach for intestinal tuberculosis is similar to that of pulmonary tuberculosis and primarily relies on anti-tuberculosis drug therapy. According to the World Health Organization (WHO) recommendations, the treatment regimen for pediatric tuberculosis includes medications such as isoniazid, rifampicin, pyrazinamide, and ethambutol. The recommended dosages are isoniazid 10–15 milligrams/kilogram, rifampicin 10–20 milligrams/kilogram, pyrazinamide 30–40 milligrams/kilogram, and ethambutol 15–25 milligrams/kilogram. The treatment duration consists of an initial intensive phase of two months followed by a continuation phase of four months to ensure the eradication of Mycobacterium tuberculosis [22]. For cases of multidrug-resistant tuberculosis, linezolid has been proven to be an effective treatment option with bactericidal activity and the ability to penetrate the cerebrospinal fluid. However, its use is limited by toxicity and the need for long-term monitoring. Therefore, further research is required to determine the optimal dosage and duration of treatment to achieve the best efficacy-toxicity balance [23]. To ensure the complete eradication of tuberculosis (TB), we adopted a long-course anti-tuberculosis treatment lasting for a total of 9 months. This treatment regimen consists of an initial intensive phase, which includes 1 month of isoniazid 100 mg qd, rifampicin 125 mg qd, and ethambutol 85 mg tid, followed by a maintenance phase that includes isoniazid 100 mg qd and rifampicin 125 mg qd.

To achieve the best therapeutic outcome, it is crucial to adhere to the full duration of the treatment course. The extended treatment duration aims to effectively eliminate TB infection and prevent the development of drug resistance. Close monitoring and regular follow-up are essential to evaluate the response to treatment and address any potential adverse effects.

Although pharmacologic anti-tuberculosis can treat most patients with intestinal TB, surgical treatment is necessary when severe complications such as intestinal obstruction, intestinal adhesions, and intestinal degeneration due to intestinal tuberculosis occur [24]. In patients with abdominal tuberculosis who have developed severe complications, the indications for surgery can be relaxed, and surgery should be performed as early as possible [25].

In our case, the child had developed severe complications. The etiological diagnosis was unclear, so we performed a laparoscopic exploration of the child. However, we found extensive intra-abdominal adhesions intraoperatively, which were challenging to separate laparoscopically, and we decided to convert to open surgery. The etiology of the abdominal infection in this child was unknown, and the possibility of abdominal tuberculosis infection was considered in combination with his low weight, hypothermia, and combined with his family history. We decided to treat the child with diagnostic anti-tuberculosis treatment with signed consent from his family. Finally, pathological sections confirmed intestinal tuberculosis infection to support our decision. After 11 days of anti-tuberculosis treatment, the patient’s condition stabilized, and they were successfully discharged. Follow-up visits were conducted for a total of 8 months after discharge, during which the patient’s general condition recovered satisfactorily, and their weight increased to 11.7 kg (below 1 SD for age). The cystostomy tube was removed 3 days after discharge. We did monthly follow-ups for the patient there was no liver function impairment. The patient developed an adhesive intestinal obstruction in the 6th month postoperatively. Fasting, rehydration, and supportive treatments were conducted for five days, the patient was then in remission and then discharged. The current treatment plan is to evaluate whether to discontinue anti-tuberculosis treatment after 9 months. The ileostomy closure was initially proposed one year after.

## Conclusion

In conclusion, the case presented here is a rare case of umbilical-enteric fistula with bladder ileal fistula secondary to intestinal perforation from intestinal tuberculosis. this article aims to make the surgical community aware of this atypical presentation of intestinal tuberculosis so that they can be prepared in case they encounter it in the future.

## Data Availability

The datasets for this article are not publicly available due to concerns regarding participant/patient anonymity. Requests to access the datasets should be directed to the corresponding authors.

## Abbreviations

BCG: Bacillus Calmette Guerin
CRP: C-reactive protein
CT: Computed Tomography
CECT: Contrast-enhanced computed tomography
ESR: Erythrocyte sedimentation rate
HIV: Human Immunodeficiency Virus
Hpf: High Power Field
H&E stain: Hematoxylin-Eosin staining
IGRA: Interferon-gamma release assay
IL-8: Interleukin-8
ITB: Intestinal tuberculosis
LTBI: Latent tuberculosis infection
PPD: Purified protein derivative
Qd: Quaque die
SD: Standard deviation
TB: Tuberculosis
Tid: Ter in die
T-SPOT: T-SPOT.TB
X-pert: X-pert MTB/RIF(Mycobacterium Tuberculosis/ Rifampicin)
